# Endoscopic endonasal approach for tuberculum sellae meningioma with optic canal involvement: standard technique for optimal visual outcomes

**DOI:** 10.3171/2025.10.FOCVID25176

**Published:** 2026-01-01

**Authors:** Yuki Shinya, Masahiro Shin, Hirotaka Hasegawa, Taichi Kin, Kenji Kondo, Nobuhito Saito

**Affiliations:** 1 Department of Neurosurgery, University of Tokyo Hospital, Tokyo, Japan;; 2 Department of Neurological Surgery, University of Virginia, Charlottesville, Virginia;; 3 Department of Neurosurgery, Teikyo University Hospital, Tokyo, Japan;; 4 Department of Neurosurgery, Saitama Medical Center, Saitama Medical University, Saitama, Japan; and; 5 Department of Otorhinolaryngology, University of Tokyo Hospital, Tokyo, Japan

**Keywords:** endoscopic endonasal approach, tuberculum sellae meningioma, optic canal involvement, visual outcome, skull base surgery

## Abstract

Anterior skull base meningiomas often cause visual decline by compressing the optic apparatus. The authors present a tuberculum sellae meningioma with optic canal involvement treated via an endoscopic endonasal approach (EEA). Early devascularization, stepwise detachment, and sharp dissection preserved surrounding neurovascular structures and perforating arteries. Optic canal drilling enabled complete removal, and multilayer reconstruction ensured watertight closure. Gross-total resection was achieved without complications. Visual acuity improved from 0.7 to 1.2, with resolution of visual field defect. This case highlights the EEA as a standard technique for optimal visual outcomes in anterior skull base meningiomas with optic canal involvement.

The video can be found here: https://stream.cadmore.media/r10.3171/2025.10.FOCVID25176

## Transcript

This video demonstrates an "Endoscopic endonasal approach for tuberculum sellae meningioma with optic canal involvement: standard technique for optimal visual outcomes."

### 0:33 Patient Presentation.

A 35-year-old man presented with a chief complaint of progressive visual disturbance over a 5-month period. On neurological examination, visual acuity of the left eye was reduced to 0.7, with a temporal hemianopsia. The patient was otherwise neurologically intact and fully independent. The patient’s past medical and family histories were unremarkable.

### 1:03 Radiological Findings.

Brain MRI demonstrated a vividly enhancing suprasellar mass, measuring 26 × 23 mm, consistent with a tuberculum sellae meningioma. The tumor, although located in the midline, was slightly displaced to the left, causing marked compression of the left optic nerve and extending into the left optic canal.

### 1:28 Three-Dimensional–Based Approach Selection.

Three-dimensional imaging was reconstructed to determine the optimal surgical corridor. The analysis indicated that the endoscopic endonasal approach would permit safe resection, especially facilitating decompression of the left optic canal and removal of the tumor infiltrating this area.

### 1:50 Surgical Setup.

The patient was placed under general endotracheal anesthesia in the supine position, with the head elevated 10° and rotated slightly toward the operator. The procedure was performed by a single surgeon using a robotic endoscope holder. A 4-mm endoscope with 0°, 30°, and 70° lenses was employed. Preoperative Stealth navigation guided the surgical corridor, and visual evoked potential (VEP) monitoring was used throughout the procedure.

### 2:25 Exposure of the Sellar Floor and Planum Sphenoidale.

The tumor was approached through a binostril endoscopic endonasal route. The sphenoid sinus was entered first, and the sellar floor extending to the planum sphenoidale was exposed. Under a bloodless field, this bony surface was carefully drilled.

### 2:45 Coagulation of Feeding Arteries: Devascularization.

Once the dura overlying the tumor was identified, one of the advantages of this approach—meticulous coagulation of the tumor’s feeding arteries, posterior ethmoidal arteries in this case—was employed.

### 2:56 Dural Opening and Tumor Exposure.

Subsequently, the dura is incised. At this stage, it is evident that the tumor has already been adequately devascularized, which facilitates its descent into the surgical field.

### 3:20 Tumor Detachment and Internal Decompression.

During tumor detachment, the initial step is to carefully identify the interface between the tumor and the preserved arachnoid membrane. As the arachnoid at the anterior skull base was intact, the margin between the tumor and the arachnoid was subsequently secured. We then proceed with internal decompression of the tumor. In cases where the tumor is soft, it often tends to spontaneously protrude into the extradural space during detachment and decompression.

### 3:57 Tumor Dissection From Critical Structures and Perforating Vessels.

Once adequate internal decompression has been achieved, dissection can proceed between the tumor and the critical neurovascular structures. In this case, a favorable dissection plane was identified between the right optic nerve and the tumor, allowing dissection to be advanced toward the optic chiasm. Further dissection is carried out between the frontal lobe, the frontobasal artery, and the tumor, and portions that have been completely detached are removed. Dissection is once again advanced from the right optic nerve toward the optic chiasm and the pituitary stalk. Particular attention is given to identifying and preserving the right frontobasal artery, the anterior communicating artery (A-com) complex, and the optic branch of the superior hypophyseal artery. Blunt dissection proved effective; however, as in conventional microsurgery, the use of endonasal scissors enabled sharp dissection and facilitated optimal exposure. With these meticulous steps, tumor removal is gradually advanced, eventually exposing the optic chiasm and the pituitary stalk.

### 5:26 Optic Canal Opening and Complete Tumor Removal.

From the optic chiasm to the left optic canal, firm adhesion was encountered. The left optic canal was opened, and the infiltrating tumor was dissected carefully and removed to achieve adequate decompression of the left optic nerve, allowing complete removal of the infiltrating tumor within this region.

### 5:50 Final View: Complete Resection With Preservation of Critical Structures.

This is the final view after complete tumor removal. The optic chiasm, optic nerves, and pituitary stalk are clearly preserved, with intact arachnoid planes and well-preserved perforating vessels. The left optic canal was also opened, and the infiltrating tumor within the canal was completely removed.

### 6:15 Skull Base Reconstruction.

A Simpson grade I–equivalent resection has been achieved, as the dural attachment and involved optic canal were addressed, although the full dural floor could not be directly visualized. Reconstruction was then performed in a multilayered fashion. DuraGen was placed, followed by inlay and onlay fascia, with fat packing supported by a sinus balloon. The bony septum was repositioned, the intranasal structures were completely restored to their original position, and the procedure was completed uneventfully.

### 7:00 Postoperative Course.

Postoperative MRI confirmed gross-total resection of the tumor. The postoperative course was uneventful, with no CSF leakage or other complications. Histopathological examination revealed a meningothelial meningioma with a Ki-67 index of 3%. Although the tumor extended partially beyond the left internal carotid artery, its soft consistency allowed sufficient internal decompression, enabling the capsule to infold and be mobilized medially. Thus, gross-total resection was feasible, and such lateral extension does not always represent a contraindication for the endonasal route in selected soft tumors. The patient’s visual acuity improved from 0.7 to 1.2, and the preoperative visual field defects completely resolved by 5 months after surgery.

### 8:08 Summary.

In summary, the endoscopic endonasal approach enabled early devascularization, safe dissection, and complete removal of the tumor, with meticulous multilayer reconstruction, ensuring watertight closure. Gross-total resection was achieved without complications, and the patient experienced significant visual recovery. While the EEA provides the higher chance of visual improvement, tailored selection between endonasal and transcranial routes remains essential depending on tumor location and vascular involvement.^1–10^ This case highlights that the EEA is a safe and effective standard technique for midline anterior skull base meningiomas when combined with expert reconstruction.

## Acknowledgments

A generative artificial intelligence program (ChatGPT 5) was used to check grammar, spelling, and language clarity in the creation of the text. No content or data analysis was generated using AI, and the intellectual content, research, and interpretations are entirely our responsibility.

## Disclosures

The authors report no conflict of interest concerning the materials or methods used in this study or the findings specified in this publication.

## Author Contributions

Primary surgeon: Shinya. Assistant surgeon: Shin. Editing and drafting the video and abstract: Shinya, Kondo. Critically revising the work: Shinya, Shin, Kondo. Reviewed submitted version of the work: Shinya, Shin, Hasegawa, Kondo, Saito. Approved the final version of the work on behalf of all authors: Shinya. Supervision: Shinya, Shin, Hasegawa, Kin.

## Supplemental Information

### Patient Informed Consent

The necessary patient informed consent was obtained in this study.
